# Functional implications of NHR-210 enrichment in *C. elegans* cephalic sheath glia: insights into metabolic and mitochondrial disruptions in Parkinson's disease models

**DOI:** 10.1007/s00018-024-05179-2

**Published:** 2024-05-01

**Authors:** Rohil Hameed, Anam Naseer, Ankit Saxena, Mahmood Akbar, Pranoy Toppo, Arunabh Sarkar, Sanjeev K. Shukla, Aamir Nazir

**Affiliations:** 1 Division of Neuroscience and Ageing Biology, CSIR-Central Drug Research Institute, Lucknow, Uttar Pradesh 226031 India; 2 Sophisticated Analytical Instrument Facility and Research, CSIR-Central Drug Research Institute, Lucknow, 226031 India; 3 Academy of Scientific and Innovative Research (AcSIR), Ghaziabad, 201002 India

**Keywords:** CEPsh glia, *nhr-210*, *pgp-9*, RNAi, HR-MAS NMR, Metabolomics

## Abstract

Glial cells constitute nearly half of the mammalian nervous system's cellular composition. The glia in *C. elegans* perform majority of tasks comparable to those conducted by their mammalian equivalents. The cephalic sheath (CEPsh) glia, which are known to be the counterparts of mammalian astrocytes, are enriched with two nuclear hormone receptors (NHRs)—NHR-210 and NHR-231. This unique enrichment makes the CEPsh glia and these NHRs intriguing subjects of study concerning neuronal health. We endeavored to assess the role of these NHRs in neurodegenerative diseases and related functional processes, using transgenic *C. elegans* expressing human alpha-synuclein. We employed RNAi-mediated silencing, followed by behavioural, functional, and metabolic profiling in relation to suppression of NHR-210 and 231. Our findings revealed that depleting *nhr-210* changes dopamine-associated behaviour and mitochondrial function in human alpha synuclein-expressing strains NL5901 and UA44, through a putative target, *pgp-9*, a transmembrane transporter. Considering the alteration in mitochondrial function and the involvement of a transmembrane transporter, we performed metabolomics study via HR-MAS NMR spectroscopy. Remarkably, substantial modifications in ATP, betaine, lactate, and glycine levels were seen upon the absence of *nhr-210*. We also detected considerable changes in metabolic pathways such as phenylalanine, tyrosine, and tryptophan biosynthesis metabolism; glycine, serine, and threonine metabolism; as well as glyoxalate and dicarboxylate metabolism. In conclusion, the deficiency of the nuclear hormone receptor *nhr-210* in alpha-synuclein expressing strain of *C. elegans*, results in altered mitochondrial function, coupled with alterations in vital metabolite levels. These findings underline the functional and physiological importance of *nhr-210* enrichment in CEPsh glia.

**Graphical abstract:**

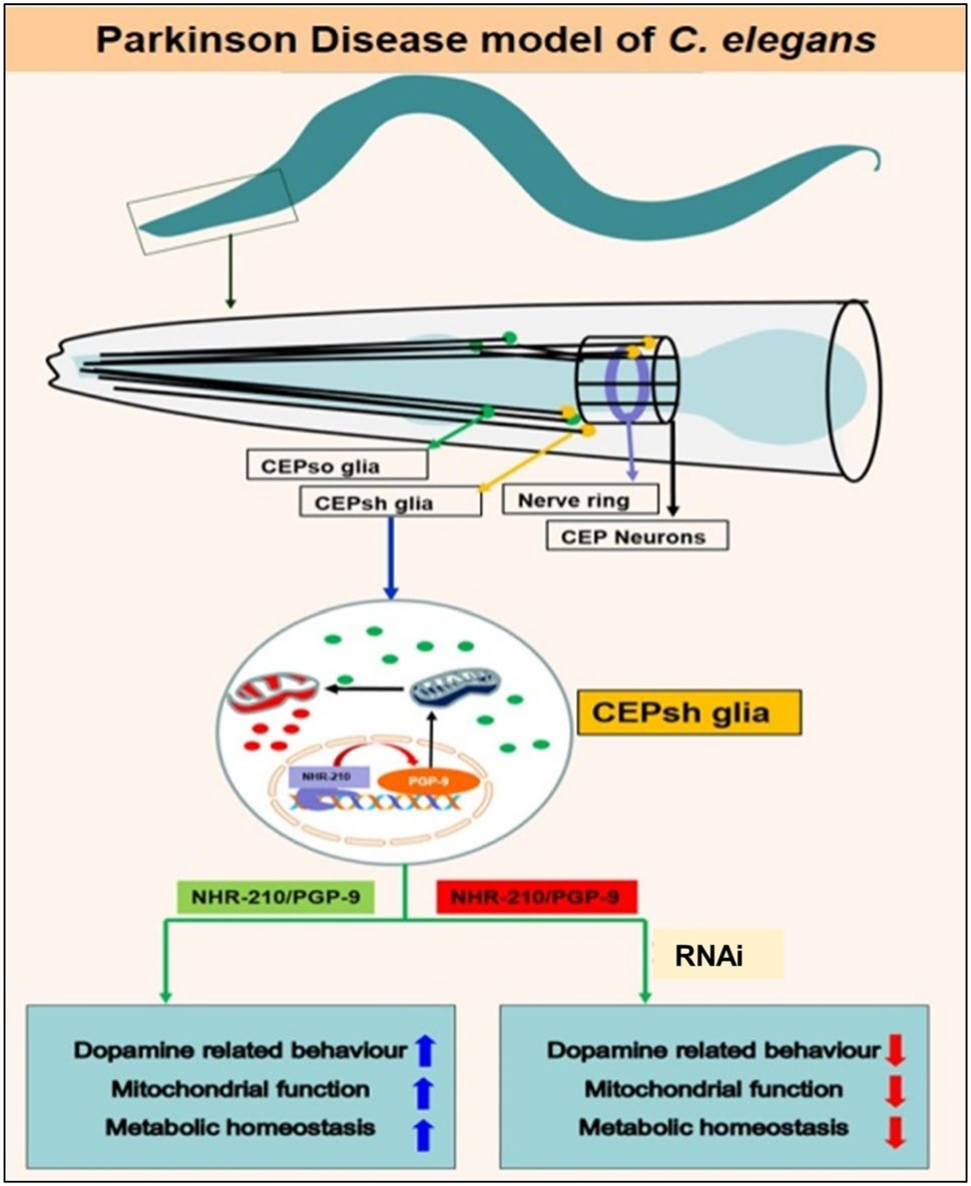

## Introduction

The development and functionality of the nervous system are intricately linked to the role of glial cells. In the model organism *Caenorhabditis elegans* (*C. elegans*) which has 302 and 56 glial cells, the cephalic sheath (CEPsh) glia in particular, are pivotal for understanding glial-neuronal interactions. [1]. Interestingly, these CEPsh glia have provided a significant understanding of the glia-neuron relationship at the molecular level, which has been translated to higher vertebrates [2]. A study by Katz et al*.* (2019) reported how presynaptic activation of MGL-2/mGluR5 triggers repetitive behaviour, because of glutamate spillover, in *C. elegans* [3]. Based on hierarchical clustering along with homolog search, a comparative analysis of CEPsh enriched genes with that of different murine brain cells revealed that CEPsh glial cells have a group of enriched genes, which include *fmi-1, glt-1, nhr-210, nhr-231, clc-1,* and *ifa-4* that have homolog in murine astrocytes [3]. Therefore, the fact that CEPsh glia are enriched with two nuclear hormone receptors (NHRs) NHR-210 and NHR-231, makes CEPsh glia as well as these NHRs interesting to study in terms of neuronal health [3].Considering the immense relevance of nuclear hormone receptors in providing a feedback loop for adaptation to changing sensory information therefore puts forth their scientific relevance in the context of neuronal health, damage, and repair.

The landmark hypothesis by Oliver Hobert [4], that the nuclear hormone receptors (NHRs) are used for adaptation to the dynamic sensory environment and thus function as sensory receptors, led to the development of novel approaches to understanding the role of these receptors in *C. elegans.* The *C. elegans* genome encodes 267 (C4-zinc finger) nuclear hormone receptors, with high sequence divergence in the *nhr-*family genes [5–7]. An intriguing correlation exists between GPCR and NHR genes, suggesting a role for *nhr* genes in sensory mechanisms. NHRs in *C. elegans* are known to be involved in a variety of functions including immune response, lipid metabolism and age regulation [8–10].

Our research focuses on the functional implications of NHR-210 in the glial cells. We hypothesize that NHR-210 plays a significant role in the healthy and diseased states of neurons, particularly in the context of neurodegenerative diseases like Parkinson's disease. To test this hypothesis, we used a transgenic *C. elegans* model expressing human alpha-synuclein, a protein associated with Parkinson's disease. Through reverse genetics approaches, including RNA interference (RNAi), we investigated how altering NHR-210 affects mitochondrial function, dopamine-related behavior, and the interaction with a key transmembrane transporter, pgp-9. Our study also extends to metabolic profiling using High-Resolution Magic Angle Spinning (HR-MAS) Nuclear Magnetic Resonance (NMR) spectroscopy. This approach allows us to detect changes in key metabolites and understand the broader metabolic consequences of NHR-210 manipulation. By examining the impact of NHR-210 on cellular and metabolic processes linked to neurodegeneration, we aim to shed light on potential mechanisms underlying PD and identify novel therapeutic targets.

Our research, hence, aims to elucidate the role of NHR-210 in CEPsh glia, offering insights into its significance in neurodegenerative conditions. This work not only enhances our understanding of *C. elegans* glial biology but also provides valuable perspectives on the broader functions of astrocyte-like cells in neurological health and disease.

## Materials and methods

### *C. elegans* culture

*C. elegans* strains were cultured on nematode growth media having lawns of *E. coli* OP-50 strain bacteria that were incubated at 22 °C [11]. Synchronized worms were obtained by treating gravid adult-stage worms with an axenising solution (4% sodium hypochlorite solution) followed by washings using M9 buffer. In this study, we employed the following strains: N2 (Bristol-wild type), NL5901 ([unc-54p::alpha-synuclein::YFP + unc-119( +)]) expressing human alpha-synuclein, OS1914 (HLH::GFP), OS2260 (PTR-10::RFP), UA44 (baIn11 [pdat-1αSyn, pdat-1gfp]) expressing human alpha-synuclein in dopaminergic neurons, RB1693 (*ptr-10* (ok2106) I) which is a *ptr-10* gene knockout and VC486 (*hlh-17* (ok487) IV/nT1 [qIs51] (IV;V)) which is *hlh-17* gene knockout. *C. elegans* strains were procured from the Caenorhabditis Genetics Centre (University of Minnesota). Strains OS2260 and OS1914 were generously provided by Prof. Shai Shaham, The Rockefeller University, USA.

### RNAi based gene silencing

We followed the standard feeding technique of RNAi for gene knockdown [12]. Since *C. elegans* is a bacterivore, we used HT115 strain of *E. coli* bacteria carrying the plasmid expressing double-stranded RNA complementary to the target gene of interest for silencing. (source: Ahringer RNAi library). The commonly used vector for this includes L4440, thus, when the worm feeds on bacteria having plasmid/clone of interest gets into *C. elegans* system and interacts with the gene of interest and silences it via RNAi phenomenon. For this study, the RNAi clones were grown independently, in LB medium having 50 μg/ml ampicillin, followed by incubation for 6–8 h at 37 °C, [11]. Bacterial clones were seeded onto NGM plates having 5 mM-IPTG (isopropylthio-beta-d-galactoside), which is used for the siRNA induction with carbenicillin (25 mg/l). The RNAi seeded plates were used after 10–12 h incubation at 22 °C. Moreover, it is already reported that the knockdown of *C. elegans* glial cell specific genes can be performed through systemic and standard RNAi method [13, 14].

### Recording and quantification of photomicrographs

Synchronized worm populations were washed three times with M9 buffer and immobilized with 100 mM sodium azide (Sigma, Cat No. 71289) at the L4 stage [12]. Immobilized worms were placed on a glass slide, which was then covered with a coverslip. To record images, we used a Carl Zeiss Axio Imager M3 microscope and ZEN2010 image acquisition software. We have used the same procedure for the measurement of worm body dimensions, through distance tool in Zeiss software. Photomicrographs (10 × and 20 × magnification) were analyzed using Image J software from the National Institutes of Health in Bethesda, Maryland. The analyzed area was kept uniform across all experimental conditions. To calculate the values for mean fluorescence intensity and for statistical analysis, we employed the GraphPad Prism 5 software.

### Thrashing assay

The pattern of thrashing in *C. elegans* gives a measure of its healthy muscular and neurobehavioral function, hence the thrashing rate was studied, which directly reveals the amount of dopamine accessible in the worms under different genetic manipulations and test conditions [11]. Worms were reared on RNAi-treated plates for 48 h in this test, since the RNAi treatment via oral administration is done at embryo stage and it requires 48 h to reach the stage being studied, that is L4/YA stage. Following that, a few worms were put in M9 buffer on a glass slide and allowed for a short period of time to acclimate. After acclimation, a stereo zoom microscope was used to examine the frequency of sigmoidal bends in each individual worm for 30 s manually. Each sample was counted 30 times, and the statistical significance of the data was determined using the student’s *t*-test.

### Isolation of total RNA and cDNA synthesis

Worms were washed thrice with M9 buffer and then with DEPC (Sigma Cat. No. D5758) treated water to extract total RNA from the worm strains (L4 stage) with approximately 100 µl worm pellet. For isolation of total RNA, we used RNAzol® RT (Sigma, Cat. No. R4533) as per manufacturer guidelines and utilized the Nanodrop (Thermo-Q5000) to determine the RNA concentration. We used1 ug of the total RNA to synthesise cDNAby cDNA synthesis kit (Thermo Fisher: AB1453B) as per manufacturer's protocol [11].

### Quantitative real-time PCR assay

The quantitative evaluation of amplified cDNA was carried out using SYBR Green (TAKARA:RR420) dye following manufacturer's protocol. For the amplification, 100 ng of cDNA was employed, and the thermal cycler (Agilent Technologies- MX3005P) device was used for the detection. We used the following amplification conditions: pre-incubation one cycle for 2 min at 50 °C and 10 min at 95 °C, followed by 40 amplification cycles for 30 s at 95 °C, 30 s at 55 °C, and 30 s at 60 °C) and to create melting curve we used 95 °C for 5 s and 65 °C for 1 min. The quantitative real-time PCR was performed at least three times, with duplicates each time. We used an endogenous control gene (*act-1*) to standardize each gene's Ct levels for analysis [15].

**Table:** 

act-1	Forward Primer: TTACTCTTTCACCACCACCGCTGA Reverse Primer: TCGTTTCCGACGGTGATGACTTGT
fmi-1	Forward Primer: CACGTGTGTTCCTGGTTACT Reverse Primer: CGGCATCGTTCATCTGTATCT
nhr-210	Forward Primer: CAAAGTGGCTGACGCATTTC Reverse Primer: GACCAGACATGCCAAGTAGATT
nhr-231	Forward Primer: CCACTATCAAGTTTGGCAG Reverse Primer: CATGAATTGGGAGCCCTCTAA
clc-1	Forward Primer: CCACTATCAAGTTTGGCAG Reverse Primer: CCACTATCAAGTTTGGCAG
glt-1	Forward Primer: CGCCAGATACACGGATTAACA Reverse Primer: CGGAGCAGGAAACCACATAG
pgp-9	Forward Primer: TACCGGAAATCGATGCATACTC Reverse Primer: CACGTGTCGGGTACGTAAAT
nhr-106	Forward Primer: TGTGAACTTAGGCACCACTAAA Reverse Primer: ATCATCGTCAATGCCGTATAGAA
K10D11.5	Forward Primer: GGACGGTTAGTATGGATGGAAC Reverse Primer: CCAGACCCAGATGCACTATTT

### Western blotting

Synchronized L4 stage worms (300 µl worm pellet) were washed three times with M9 buffer and re-suspended in worm lysis buffer (20 mM potassium phosphate, 2 mM EDTA, 1% Triton X 100, 1 × Protease K) [11]. Worms were sonicated at 25 amplitude for 3 min (15 s pulse time on and off) using a Thermo Scientific Sonicator. The sonicated lysate was centrifuged at 13,000 rpm for 20 min at 4 °C, to collect the total worm protein in the supernatant. The protein concentration was quantified using Bradford’s reagent. For each group, 30 µg of protein was loaded onto a 10–12% sodium dodecyl sulfate–polyacrylamide gel electrophoresis. The primary antibodies used for western blot analysis were diluted 1:1000 (Primary alpha-syn and actin antibodies with cat numbers: Millipore-MABN389 and Abcam-1801, respectively) and secondary antibodies 1:10,000 (cat number: HRP-Abcam-7090), by using 0.01% PBST. The chemiluminescence detection, western blot analysis, and densitometry analysis were performed with the standard protocol by ImageJ software.

### Mitochondrial membrane potential (ΔΨM) analysis

In order to measure the mitochondrial membrane potential, we first isolated the cells using standard protocol [16]. Briefly, L4 stage (200 μl worm pellet) worms were washed twice using M9 buffer and were re-suspended and washed twice using distilled water. The worm pellet was incubated with 200 μl of SDS-DTT (0.25% SDS, 200 mM DTT, 20 mM HEPES, pH 8.0 and 3% sucrose) solution for 4 min at RT, followed by addition of 800 μl of egg buffer (118 mM NaCl, 48 mM KCl, 2 mM CaCl_2_, 2 mM MgCl_2_, 25 mM HEPES, pH 7.3) and centrifugation at 13,000 rpm for 1 min, repeated 5 times washing was done using egg buffer, followed by 15 mg/ml Pronase treatment (Sigma-Aldrich: 10165921001) enzyme for 20 min at RT. This was followed by termination of digestion using L-15medium (Sigma-Aldrich: L1518) and centrifuged at 180*g* for 5 min at 4 °C. Cells were re-suspended in 1 ml L-15 medium and left on ice for 15 min allowing debris to settle. Supernatant was transferred into a fresh tube and centrifuged at 180*g* for 5 min at 4 °C. After cell isolation, mitochondrial membrane potential analysis was done [17]. For this, around 1 × 10^5^ cells/ml were re-suspended in the serum-free RPMI media. For staining, JC-1 dye (2.5 μg/ml, Abcam ab113850) was incubated for 15 min at RT. The MMP analysis was done using flow cytometer with excitation at 488 nm and emission at 530 and 590 nm.

### Measuring total mitochondrial content

To study the effect of genes silenced via RNAi on the total active mitochondrial content, worms were raised till L4 stage. Worm pellet of 100 μl were washed with M9 buffer thrice then 1 μl of mitotracker-DND-RED (Invitrogen) were diluted 500 μl of M9 buffer separately. In the prepared diluted dye around 500 worms were incubated for around 1 h at 22 °C (light protected tube). After the incubation worms were washed thrice using 1 ml M9 buffer to remove excesses dye followed by anesthetised using sodium azide and finally mounted on glass slide for the microscopic analysis [18].

### Acridine orange staining

To study the effect of RNAi of *pgp-9* on apoptosis, we employed NL5901 strain of *C. elegans* using previously described method [19]. Briefly, worms were raised till L4 stage on respective RNAi treated plates, followed by wishing with M9 buffer thrice. Acridine orange stock solutions were prepared 10 mg/ml and 7.5 μl were diluted in 1 ml of M9 buffer. We used 200 μl worm pellet followed by treatment with 500 μl of diluted acridine orange for 1 h in the dark and washed with M9 buffer thrice (de stain excess AO). Finally, worms were anesthetised using sodium azide and finally mounted on glass slide for the microscopic analysis.

### HR-MAS NMR evaluations

To probe the metabolic shifts triggered by RNAi treatment on specific genes, we employed the alpha-synuclein expressing (NL5901) and wild-type (N2) strains of *C. elegans* and carried out HR-MAS NMR based experiments. We employed a modified version of the method previously described [20], with minor adjustments tailored to our experimental needs. Each sample contained roughly 10,000 worms, and we prepared three biological replicates for each group to ensure robustness and reproducibility of our observations (hence a total of 3 ×  ~ 10,000 worms were studied). The evaluations were carried out employing a Bruker Avance II 400 MHz NMR spectrometer, operating at a proton NMR frequency of 400.13 MHz. This was equipped with a 5 mm HR-MAS 13C-1H Z gradient probe with a magic-angle gradient, and the samples were spun at a rate of 4000 ± 1 Hz, maintaining a temperature of 298 K [21]. The worm samples were stored at −80 °C and for NMR analysis, samples were thawed on ice bathtub (at 4 °C) and placed in a 4 mm zirconia rotor with 50 µl capacity, followed by adding 20 µl D_2_O (containing 0.05 wt% TSP); D_2_O serves as lock solvent and TSP as a reference [20]. The water pre saturated 1D proton Carr–Purcell–Meiboom–Gill (CPMG; sequence: cpmgpr1d, Bruker) [RD-90°-{*τ*-180°-τ}_n_ acquisition] was employed to escalate the identification of small molecular weight metabolites and suppress the macromolecular background of proteins, lipids and other substances with short T_2_ times [22]. CPMGPR1D NMR experiments were acquired with given parameters; relaxation delay 4 s; spectral sweep width 20.54 ppm; flip angle of radiofrequency pulse 90°; data points 32 K; and with 256 scans. Pulse width P1 (14 µs) and pulse power Pl9 (56.88 dB) were optimised by using command pulsecal. Data were processed (manual phase correction and baseline correction) in Chenomx (NMR suite 9.0 professional) processor module and chemical shifts were referenced concerning the TSP signal at *δ* 0.00 ppm. Residual water signal in region (4.75–4.90 ppm) was excluded from analysis in Chenomx processor module.

### 1-Nonanol assay

1-Nonanol assay is a chemotaxis assay to assess the ability of worms to avert to the smell of repellent 1-nonanol [23]. This behaviour is regulated by dopamine signalling, thus it gives qualitative measure for dopamine levels. Briefly, synchronous population of worms were reared on RNAi-treated plates for 48 h and embryos were allowed to reach L4/YA stage in order to observe the effects on chemotaxis response. Following that, a drop of 1-nonanol is brought in close proximity to the snout of the worms and its response time to avert is noted. For each group 30 readings were taken and the statistical significance of the data was determined using the student’s *t*-test.

### Data analysis

For microscopy-based quantification assays, we have performed at least two separate experiments in which we treated and analyzed more than 100 worms for each group. For each experiment, randomly selected photomicrographs are taken, as contrary to the fluorescence intensity of worms being monitored for the complete set of slides. We eventually examine the fluorescence pattern in 100–200 worms and take at least *n* = 20 photographs. Then, using Image J analysis, these photomicrographs are processed to quantify the intensity. Analysis of statistically quantified fluorescence pictures, ROS values from luminometer, the number of thrashes and worms' responses to nonanole, and the ct values acquired from qPCR procedures, we follow non-parametric independent ‘*t*’ test via Prism 5 software, where *p*-value is represented as; *p < 0.05, & ns: non-significant. We have employed L4 stage worms in all of our experiments. We used an empty vector (EV) that does not contain any dsRNA against any gene as the control groups in order to access any phenotypic, behavioural, or genetic alterations in the *C. elegans*, since it does not bring about any change in either physiology or anatomy of the worm.

## Results

### Silencing of CEPsh enriched *nhr-210* and *231* ameliorates α-synuclein expression and ROS levels

**Fig. 1:**
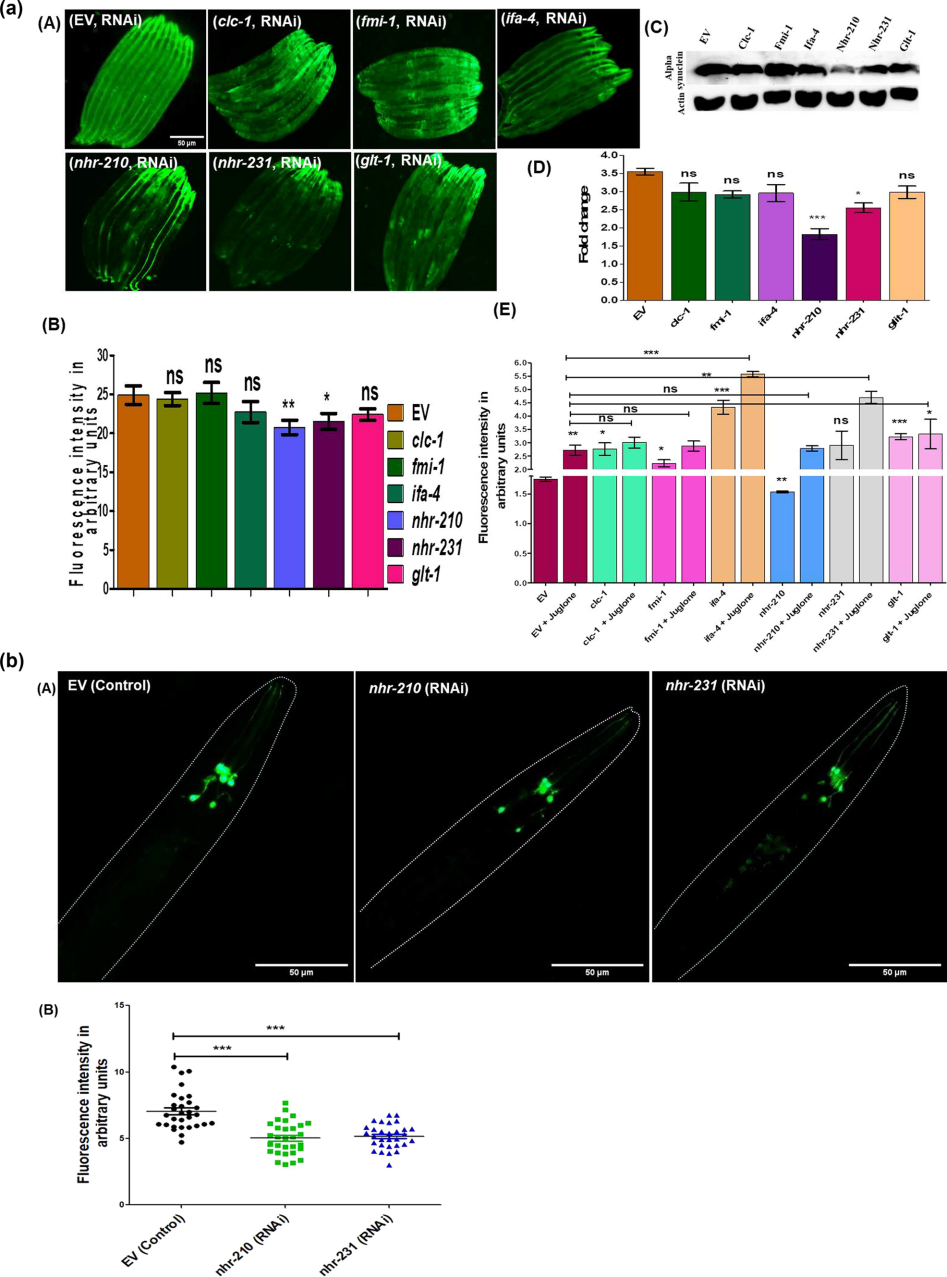
**(a) A** Representative photomicrographs of NL5901 strain of *C. elegans,* expressing human alpha-synuclein protein, control (EV), *clc-1* knockdown, *fmi-1* knockdown, *ifa-4* knockdown, *nhr-210* knockdown, *nhr-231* knockdown, and *glt-1*knockdown. **B** Column graph shows the relative fluorescence intensity in arbitrary units, *N* = 30 at 10×, scale bar 50 μm (*P-value < 0.05). **C** Representative blot showing the expression of human alpha synuclein upon the RNAi of CEPsh genes. **D** Column graph shows densitometry analysis of the representative blot (*P-value < 0.05). **E** The column graph showing ROS level upon the RNAi of CEPsh genes with and without juglone (positive control), employing N2 strain (wild type), *n* = 300, (*P-value < 0.05). **(b) A** Representative Photomicrographs of UA44 (baIn11 [pdat-1αSyn, pdat-1gfp]) strain of *C. elegans,* expressing human alpha-synuclein::GFP protein under dat-1 promoter, control (EV), *nhr-210* knockdown and *nhr-231* knockdown. **B** Representative graph shows the relative fluorescence intensity of control and knockdown conditions of *nhr-210* and *nhr-231* respectively (*N* = 30), at 20×, (*P-value < 0.05)

There has lately been an increased interest in understanding the diverse roles of NHRs, because of their nature as interconnecting molecular players in metabolite based gene regulation across the animal kingdom [24]. We applied reverse genetics approach (RNAi) to study the effect of all the noted CEPsh enriched genes on α-synuclein aggregation and ROS levels in Parkinson disease model of *C. elegans*. Upon the RNAi of CEPsh enriched genes, only *nhr-210, nhr-231* and *glt-1* showed significant decrease in α-synuclein level in NL5901 strain of *C. elegans* which expresses human alpha synuclein; however, the decrease in α-synuclein level upon *clc-1, fmi-1* and *ifa-*4 RNAi was observed to be non-significant with relative folds change of 0.5, 0.5 and 0.4, respectively. The photo micrographic analysis of fluorescence intensity around the nerve ring showed that the silencing of *nhr-210, nhr-231* and *glt-1* decreased human α-synuclein level by 4, 3.5 and 3 relative folds, respectively with respect to control (EV) (HT115 bacteria with scrambled L4440 plasmid) (Fig. 1A). Further, we validated our finding via immunoblotting; silencing of *nhr-210* and *nhr-231* showed significant decrease in the human α-synuclein expression level. Therefore, both the approaches suggest that the loss of *nhr-210* and *nhr-231* significantly decrease human α-synuclein expression levels**.** We further validated our findings by employing UA44 (baIn11 [pdat-1αSyn, pdat-1gfp]) strain of *C. elegans* having human alpha synuclein expression within the dopaminergic neurons. We observed a significant decrease in the fluorescence intensity by three relative folds upon the knock-down of *nhr-210* and *nhr-231,* respectively compared to control (EV) (Fig. 1B). These findings provide new insight into the involvement of glial cell enriched nuclear hormone receptors in neurodegenerative diseases.

Furthermore, neurodegenerative diseases are known to be associated with dysfunctional mitochondria and related molecular events [25]. Therefore, to determine the functional status of mitochondria upon the RNAi of CEPsh enriched genes (*fmi-1, glt-1, nhr-210, nhr-231, clc-1*, and *ifa-4*) we estimated ROS levels by employing wild type strain (N2) of *C. elegans*, using juglone as positive control (Fig. 1E). Our results revealed that upon the knockdown of each of the CEPsh glia enriched genes, RNAi of *nhr-210* and *fmi-1* significantly decreased the ROS levels by 0.5 relative folds with respect to control (EV). We also observed that RNAi of *clc-1, ifa-4,* and *glt-1* significantly increased ROS levels by 1, 3, and 1.5 relative folds, respectively, compared to control (EV) at baseline. We then studied the ROS levels after induction with Juglone (a ROS inducing naphthoquinone) and figured that upon the RNAi of *clc-1, fmi-1, and nhr-210,* followed by juglone treatment, there was a non-significant effect on ROS levels at the organismal level. We also observed that RNAi of *ifa-4, nhr-231, and glt-1* showed a significant increase in ROS levels in the presence of Juglone. These results suggested that the worms with RNAi of *clc-1, fmi-1, and nhr-210* lacked the antioxidant response when external ROS inducing assault was provided.

### RNAi of *nhr-210* modulates global glia enriched genes

**Fig. 2:**
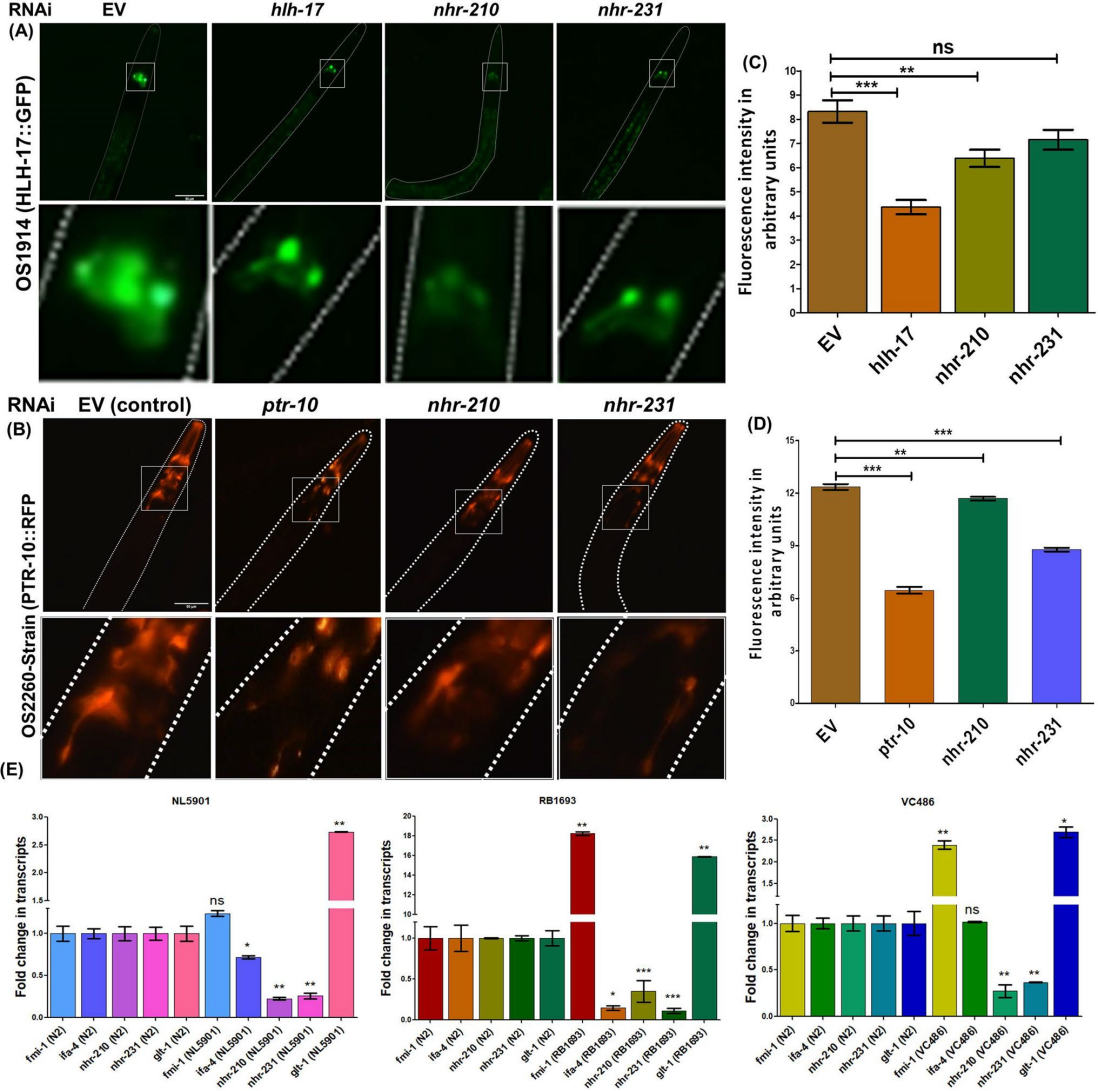
**A** Representative photomicrographs of OS1914 (*hlh-17::gfp*) strain of *C. elegans****,*** along with digitally zoomed images, Control (EV), knockdown of *hlh-17*, knockdown of *nhr-210* and knockdown of *nhr-231*. **B** Representative photomicrographs of OS2260- strain (*ptr-10:: rfp*) of *C. elegans,* along with digitally zoomed images***,*** Control (EV), knockdown of *ptr-10*, knockdown of *nhr-210* and knockdown of *nhr-231.* **C** Column graph representing relative fluorescence intensity of HLH-17::GFP upon the knockdown of *hlh-17, nhr-210*, and *nhr-231*, compare to control (EV), (*N* = 20) at 20× (*P-value < 0.05). **D** Column graph representing relative fluorescence intensity of PTR::RFP upon the knockdown of *ptr-10, nhr-210*, and *nhr-231* compare to control (EV), (*N* = 20), at 20*x* (*P-value < 0.05). **E** Representative column graph showing fold change in transcripts via RT-PCR, the expression profile of CEPsh enriched genes in NL5901, RB1693 and VC486 strains with respect to N2 strain. (*P-value = 0.05, *n* = 3)

Our studies herein demonstrate that the loss of *nhr-210* and *nhr-231*, which are enriched in CEPsh glial cells, modulates molecular events associated with Parkinson’s disease. It is known that *hlh-17* and *ptr-10* genes exhibit enriched expression within the glial cells of *C. elegans* [26]. This raises an intriguing question regarding the relationship between *nhr-210* and *nhr-231* and the glial cell-specific genes, *ptr-10* and *hlh-17*. In order to investigate effect of CEPsh glia enriched nuclear hormone receptors on global glial cell enriched gene *ptr-10* and CEPsh glia enriched gene *hlh-17,* we employed OS2260 and OS1914 strains of *C. elegans* in which PTR-10 and HLH-17 are fluorescently tagged with RFP and GFP, respectively, thereby providing a visual end-point to assess changes in their expression. In OS1914 strain the loss of *nhr-210* showed significant, three relative folds, decrease in the expression of HLH-17::GFP expression compared to control (EV). Similarly, in OS2260 strain, the RNAi mediated loss of *nhr-210 *and *nhr-231* caused significant, decrease in the PTR*-*10::RFP expression (Fig. 2A, B). Moreover, to validate these findings from the other way around, we employed the knock out strains of *ptr-10* and *hlh-17*, RB1693 and VC486 respectively, and analysed the expression level of CEPsh enriched *nhr-210* and *nhr-231.* In RB1693, the expression of *nhr-210* and *nhr-231* was found to be significantly decreased by 3 and 6 relative folds, respectively, in comparison to their expression in wild-type strain. Similarly, in VC486 their expression was found to be significantly decreased by 3.8 and 3 relative folds, respectively. Besides this, we also analysed the expression levels of *nhr-210* and *nhr-231* in NL5901 strain of *C. elegans,* showing significant decrease by 4 and 3.8 relative folds, respectively (Fig. 2E).

### CEPsh enriched nuclear hormone receptor-210 modulates worm behaviour

**Fig. 3:**
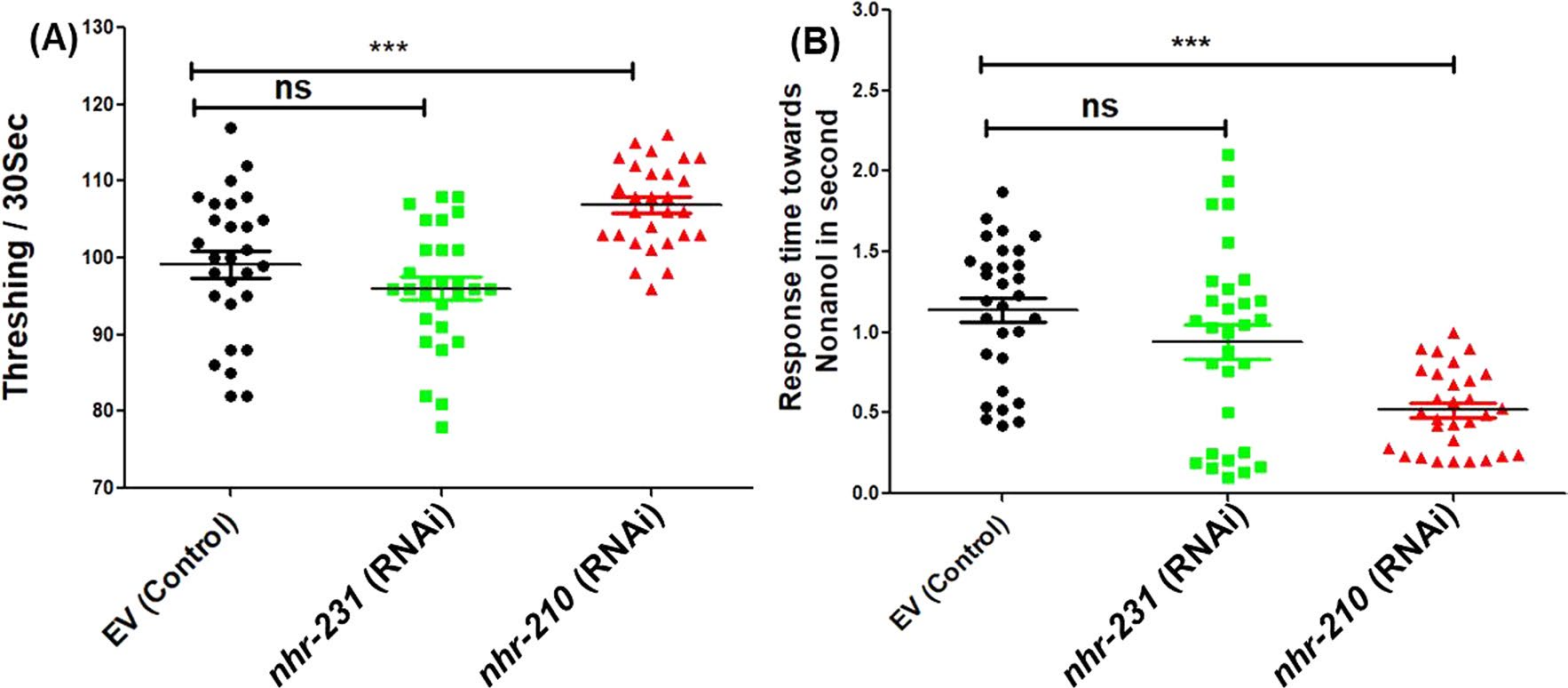
Knockdown of *nhr-210 and 231* significantly modulates worm behaviour. **A** Bar graph showing thrashing rate of NL5901 strain of *C. elegans* under knock-down conditions of *nhr-210* and *nhr-231* (*n* = 30, *P-value < 0.05). **B** The bar graph showing reaction time of NL5901 worms against nonanol upon RNAi of *nhr-210* and *nhr-231* (*N* = 30, *P-value < 0.05)

The neural architecture of *C. elegans* consists of eight dopamine-producing neurons, six of which surround the nerve ring (named CEPs and ADEs) and two are situated in the mid-body (referred to as PDEs) [27]. Given our findings indicating a modulation of *nhr-210* in the alpha-synuclein expressing *C. elegans* strain, we sought to study the effects of *nhr-210* and *nhr-231* knockdown on dopamine levels by analyzing phenotypic endpoints associated with dopamine signalling, including thrashing and chemotactic responses. In the NL5901 strain, *nhr-210* RNAi caused a significant increase in the thrashing rate, yielding a mean difference of 8.900 ± 2.775 thrashes per minute compared to the control. However, the *nhr-231* RNAi did not significantly affect the NL5901 worms' thrashing rate (Fig. 3A). We observed similar results for the nonanol aversion assay, which measures *C. elegans* response time to the repellent, nonanol, a dopamine-related function. We found that *nhr-210* RNAi significantly decreased NL5901 worms' response time to nonanol by 0.4010 ± 0.1437 s compared to the control (EV). Nevertheless, the *nhr-231* RNAi did not significantly affect the response time (Fig. 3B). These findings suggest that the CEPsh-enriched nuclear hormone receptor-210 plays a crucial role in maintaining dopamine-related functions in Parkinson's disease model at organismal level.

### Identification of predicted targets of NHR-210 and their effect on α-synuclein expression

**Fig. 4:**
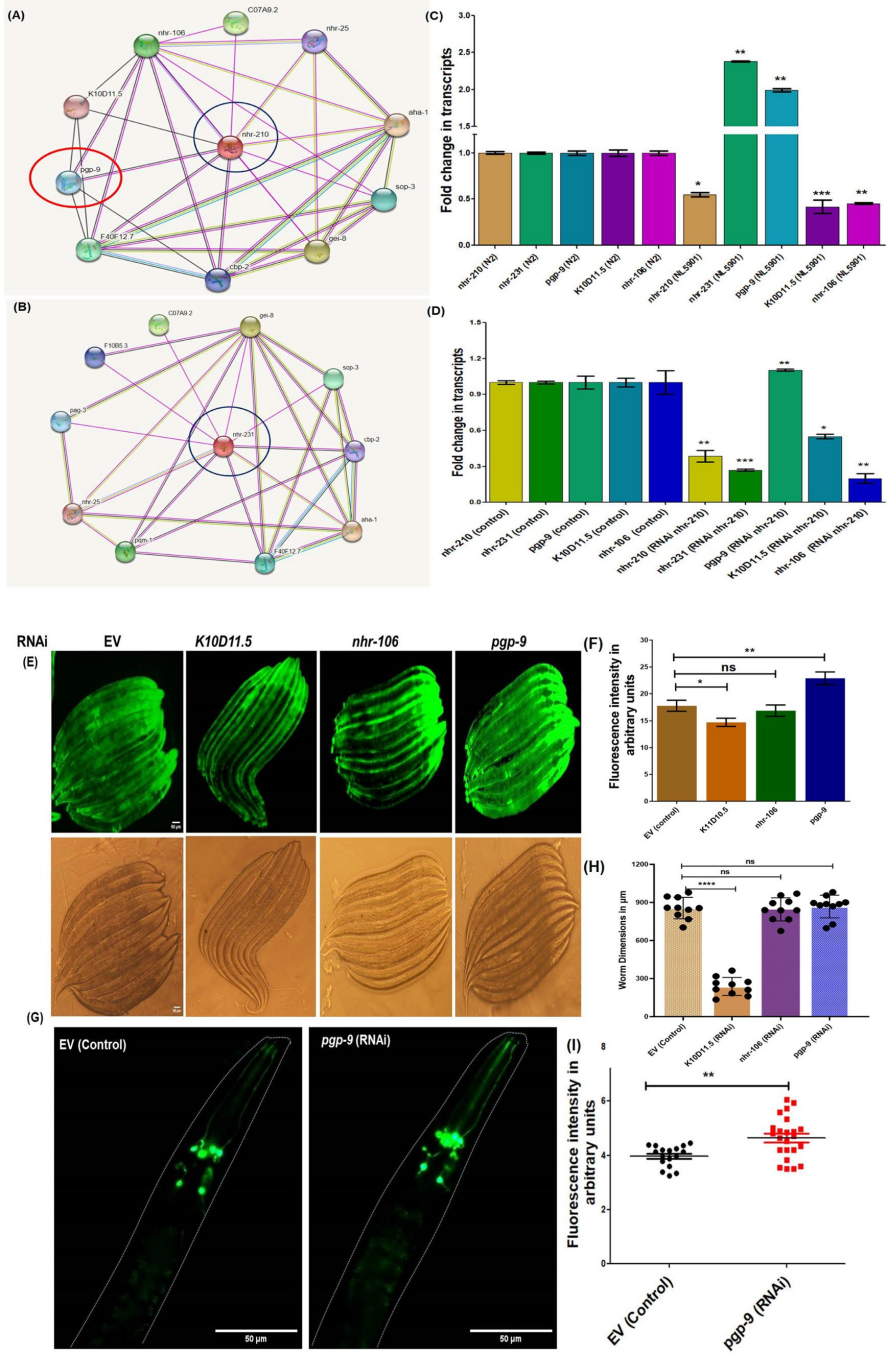
STRing based in silico target analysis of NHR-210 and NHR-231. **A**, **B** Interacting protein targets of NHR-210 and NHR-231. **C** RT-PCR based validation of predicted targets of *nhr-210*and *nhr-231 *employing N2 (wild type) vs NL5901 strain of *C. elegans* on OP-50*. ***D** RNAi of *nhr-210* increases the transcript levels of *pgp-9* in NL5901 strain of *C. elegans* (*P-value < 0.05). **E** The representative photomicrographs showing the expression of human α-syn employing NL5901 strain along with DIC images, Control (EV), *K10D11.5* RNAi *pgp-9* RNAi and *nhr-106* RNAi**,** Scale bar of 50 μm (*N* = 30)*.* **F**, **H** Column graph shows the levels of relative fold change in human alpha synuclein and worm dimensions upon the knockdown of *K10D11.5*, *pgp-9* and *nhr-106* (*P-value < 0.05). **G** The representative photomicrographs of UA44 strain of *C. elegans* upon RNAi of EV (control) and *pgp-9* (*N* = 30)*.* **I** Column graph shows the levels of relative fold change in human alpha synuclein in dopaminergic neurons upon the knockdown of EV and *pgp-9* (*P-value < 0.05)

Nuclear hormone receptors are typically identified as transcriptional factors known for regulating metabolic homeostasis [24, 28]. In this context, we endeavored to study the predicted targets of NHR-210 and NHR-231 using an in silico approach employing the STRING software. Our analysis revealed that NHR-210 and NHR-231 share six common targets, including AHA-1, GEI-8, CO7A9.2, F40F12.7, SOP-3, CBP-2, and NHR-25. We also found that NHR-210, of particular interest based on earlier results, has specific predicted targets, including PGP-9, K10D11.5, and NHR-106 (Fig. 4A, B). These findings prompted us to determine whether there were any changes in the expression of these genes in the Parkinson's disease model of *C. elegans*. To this end, we conducted real-time PCR to profile the expression of these genes. Our results clearly indicate a significant decrease in the expression of *nhr-210, k10d11.5*, and *nhr-106* by 0.5, 0.6, and 0.6 folds, respectively, compared to the wild type strain N2. Intriguingly, we also observed a significant increase in the expression of *nhr-231* and *pgp-9* by 2.5 and 2 folds, respectively.

Subsequently, we performed real-time PCR-based analysis following the knockdown of *nhr-210* in the NL5901 strain of *C. elegans*. Our data showed that the knockdown of nhr-210 led to a significant decrease in the expression of nhr-210, nhr-231, k10d11.5, and nhr-106 by 0.6, 0.7, 0.4, and 0.7 folds, respectively, relative to the control, also shows specific RNAi effect *nhr-210*. Additionally, the knockdown of nhr-210 significantly increased the expression of pgp-9 by 1.2 folds in NL5901 compared to the control (EV) (Fig. 4C, D). These results validated the in silico findings and revealed the association between these hormonal receptors. Moreover, we identified a positive correlation between *nhr-210* and *nhr-231* at the molecular level. In reference to above results our focus remained primarily on NHR-210 interacting targets. Thus, we explored the knockdown effects of the identified NHR-210 targets *pgp-9*, *k10d11.5*, and *nhr-106 *on alpha-synuclein expression. Our study demonstrated that the knockdown of *pgp-9* and *k10d11.5* altered human alpha-synuclein expression, and notably, the *pgp-9* knockdown significantly increased alpha-synuclein expression (Fig. 4E, F). We further validated our finding using the UA44 (baIn11 [pdat-1αSyn, pdat-1gfp]) strain. We observed a significant increase in human alpha-synuclein expression in the dopaminergic neurons specifically, with a two fold relative increase in fluorescence intensity upon the knockdown of *pgp-9* compared to the control (EV) (Fig. 4G, H). Although we observed that the knockdown of k10d11.5 decreases the levels of human alpha-synuclein, this knockdown also resulted in a developmental delay at L4 stage in the worms. Additionally, worms with k10d11.5 knockdown appeared leaner and had significantly reduced body dimensions with mean dimensions of 300 μm compared to control worms having mean dimensions of 900 μm. Consequently, we discontinued further investigation of the k10d11.5 gene. These findings led us to focus on *pgp-9*, as its presence is crucial for reducing the expression levels of human alpha-synuclein. Our data indicates that the knockdown of *nhr-210* increases *pgp-9* transcript levels, while the knockdown of *pgp-9* elevates human alpha-synuclein levels in both dopaminergic neurons and muscle cells. Taken together, these results suggest that *pgp-9*, as a predicted target of NHR-210, appears to operate in a regulatory loop system associated with protein aggregation. In this system, *nhr-210* modulates the expression of *pgp-9*, which in turn affects human alpha-synuclein expression at the organismal level.

### RNAi of ATP efflux transmembrane transporter ‘PGP-9’ negatively regulates mitochondrial function

**Fig. 5:**
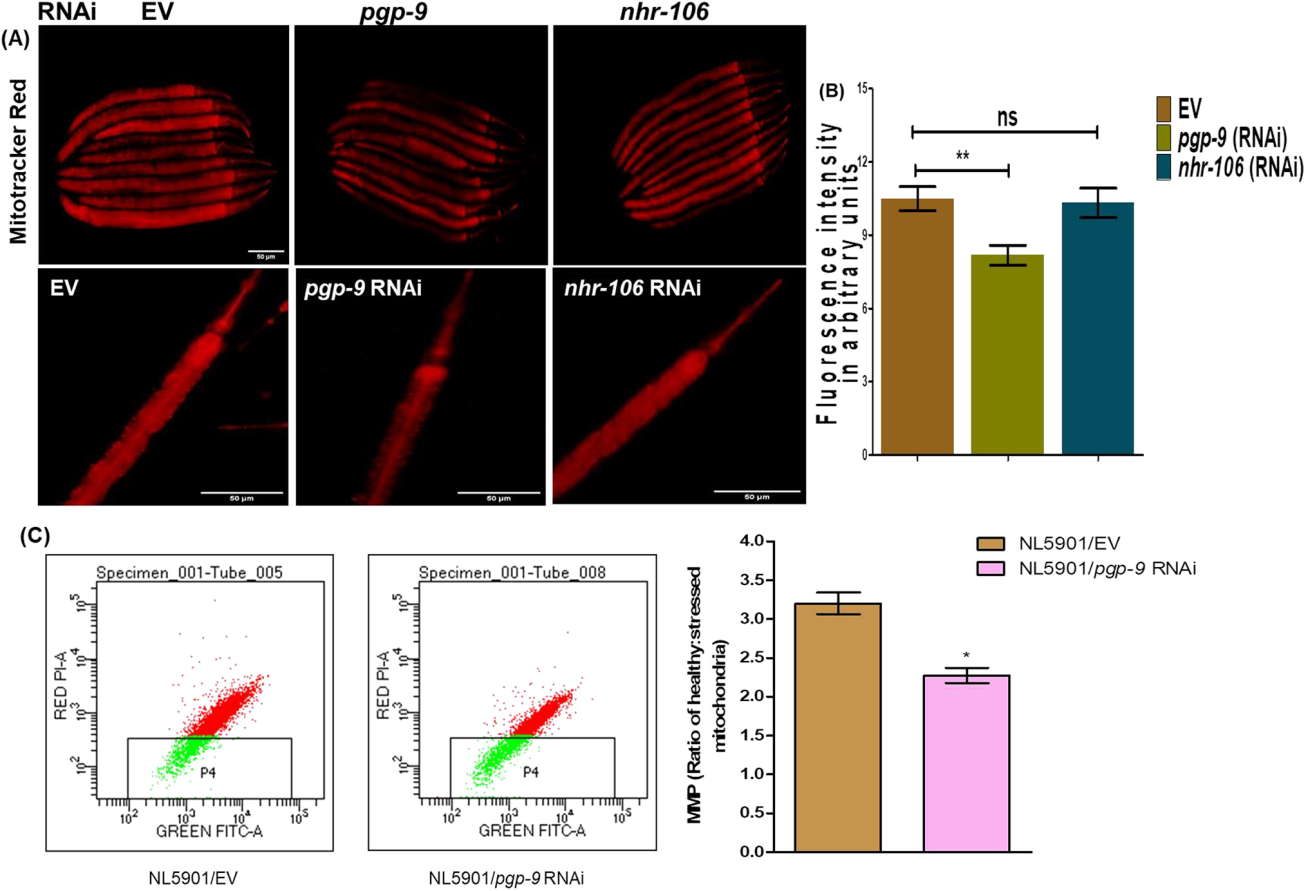
**A** Representative Photomicrographs Mitotracker stained NL5901 worms Control (EV), *pgp-9* knockdown and *nhr-106* knockdown; images were taken at 10× (*N* = 30). **B** Column graph represents relative fluorescence intensity in arbitrary units (*P < 0.05), (*n* = 30). **C** Mitochondrial membrane potential (ΔΨM) analysis. **A** Graphical representation of the ratio of healthy:stressed mitochondria. Mitochondrial depolarization is indicated by reduced red: green fluorescence ratio, as seen in case of EV and *pgp-9* RNAi (**A**, **B**). Flow cytometery result showing the region of red and green fluorescence of JC-1 dye

Our findings suggest that the nuclear hormone receptor-210 potentially modulates effects associated with neurodegenerative diseases by regulating dopamine levels and affecting alpha-synuclein expression. PGP-9, one of the predicted targets of NHR-210, exhibits ATPase-coupled transmembrane transporter activity and efflux transmembrane transporter activity (wormbase). Since numerous studies have indicated a strong link between mitochondrial dysfunction and the progression of neurodegenerative diseases, we endeavored to study the function of this ATP efflux transmembrane transporter, PGP-9, in the NL5901 strain of *C. elegans*. We knocked down *pgp-9, k10d11.5* and *nhr-106* in the NL5901 strain and analysed their effects on mitochondrial function. We first assessed the total active mitochondrial content through Mitotracker staining. The photomicrographic analysis of NL5901 worms stained with Mitotracker revealed that the knockdown of *pgp-9* significantly decreased the total active mitochondrial content, with no change observed following *nhr-106* knockdown (Fig. 5A). We then investigated whether this decrease in active mitochondria, upon the loss of *pgp-9*, was also reflected in the mitochondrial membrane potential. Our results showed that the RNAi of *pgp-9* significantly reduced the population of healthy mitochondria by inducing mitochondrial membrane depolarization. We observed a decrease in mitochondrial membrane potential (ΔΨM), indicated by a reduced red: green fluorescence ratio, in the case of *pgp-9* RNAi in NL5901 worms (Fig. 5B). Thus, our results suggest that *pgp-9* is a predicted target of *nhr-210* that regulates mitochondrial function, indicating a functional link between *nhr-210* and mitochondrial biology in Parkinson disease model of *C. elegans*.

### Loss of *pgp-9* induces apoptosis in PD model of *C. elegans*

**Fig. 6:**
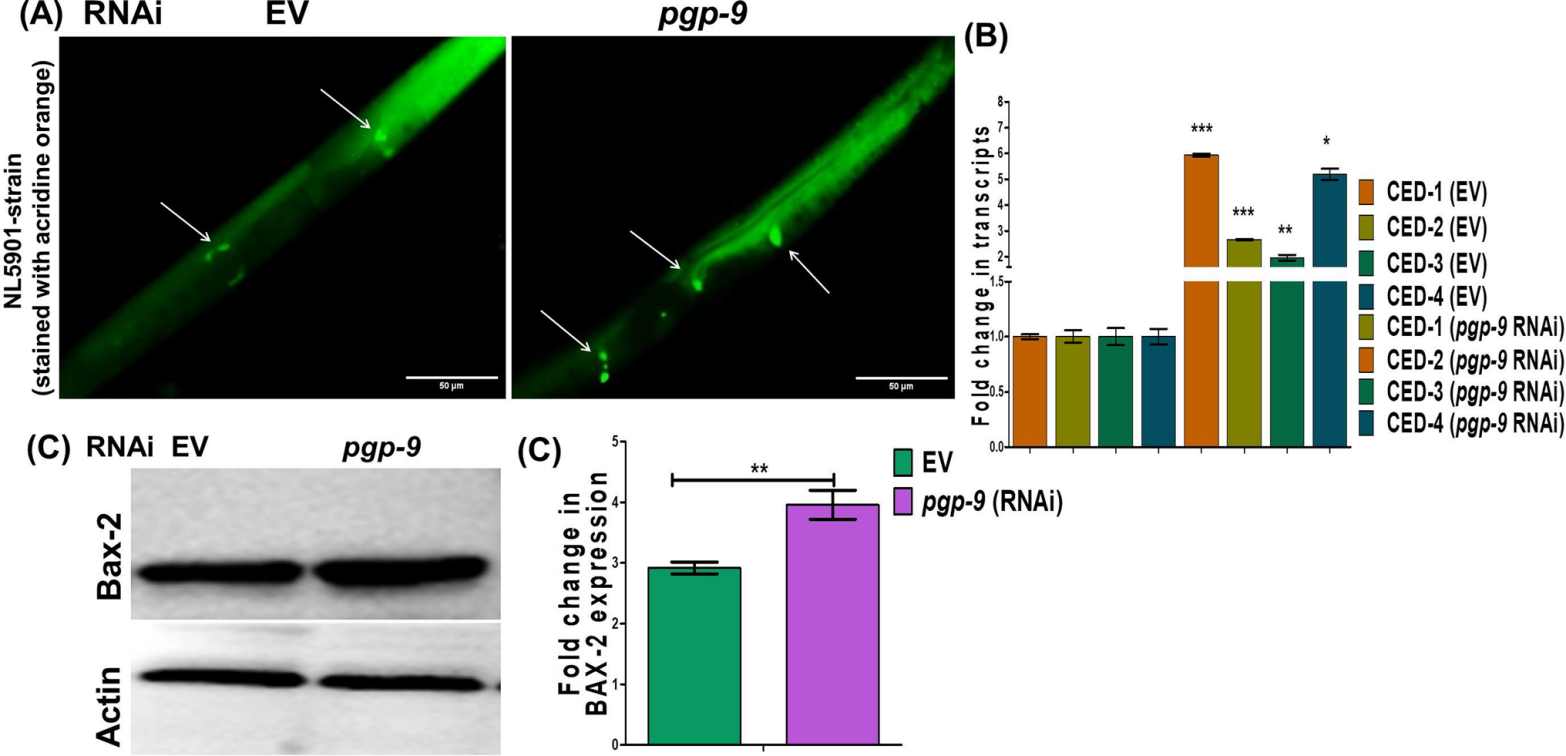
Knockdown of *pgp-9* enhanced apoptosis in NL5901 strain of *C. elegans*. **A** Representative Photomicrograph’s showing apoptotic patches (indicate by arrow) stained with acridine orange, control and *pgp-9* knockdown (*N* = 10). **B** Column graphs showing significant increase in the apoptotic marker genes analysed through real time PCR (*P-Value = 0.05). **C**, **D** Representative column graph and immuno blots showing significant increase in the expression of apoptotic marker proteins, BAX-2, stained with respective humanized antibodies

Mitochondrial dysfunction is a well-established fact in neurodegenerative disease conditions, with numerous studies investigating its causes [29, 30]. Building on our previous findings, where we suggested the involvement of *pgp-9* in normal mitochondrial functioning and its loss leading to mitochondrial dysfunction, we explored whether this loss of active mitochondria affected the homeostatic balance of apoptosis in *C. elegans*. As mitochondrial dysfunction is known to play a role in regulating cellular apoptosis, we studied the global rate of apoptosis in the NL5901 strain following the loss of *pgp-9*, compared to the control (EV). Our results showed that the loss of *pgp-9* significantly increased apoptotic patches, which were identified using the acridine orange dye (Fig. 6A). We observed the apoptotic patches around the gonad arm, both at the anterior and posterior side of the worm body. Upon detailed analysis, we noted that both the number and intensity of patches were higher in the *pgp-9* RNAi condition compared to the control. Additionally, we examined changes in the transcript level of main apoptotic marker genes following the loss of *pgp-9* in the NL5901 strain, using real-time PCR (Fig. 6B). Our analysis revealed that the knockdown of *pgp-9* significantly increased the expression of apoptosis markers, including *ced-1, ced-2, ced-3,* and *ced-4*, thus validating the results previously obtained from acridine orange-based staining. We further validated these findings through immunoblotting, demonstrating that the loss of *pgp-9* in the NL5901 strain of *C. elegans* enhanced the expression of BAX-2 compared to the control; BAX-2 is a critical regulator of apoptosis (Fig. 6C). In conclusion, our results suggest that the possible mechanism through which nuclear hormone receptor-210 operates is via *pgp-9*, which in turn regulates apoptosis, thereby modulating *C. elegans* physiology in the disease model.

### HR-MAS NMR based characterization of metabolites upon *RNAi of nhr-210* and *pgp-9*

#### Characterization of metabolites

**Fig. 7:**
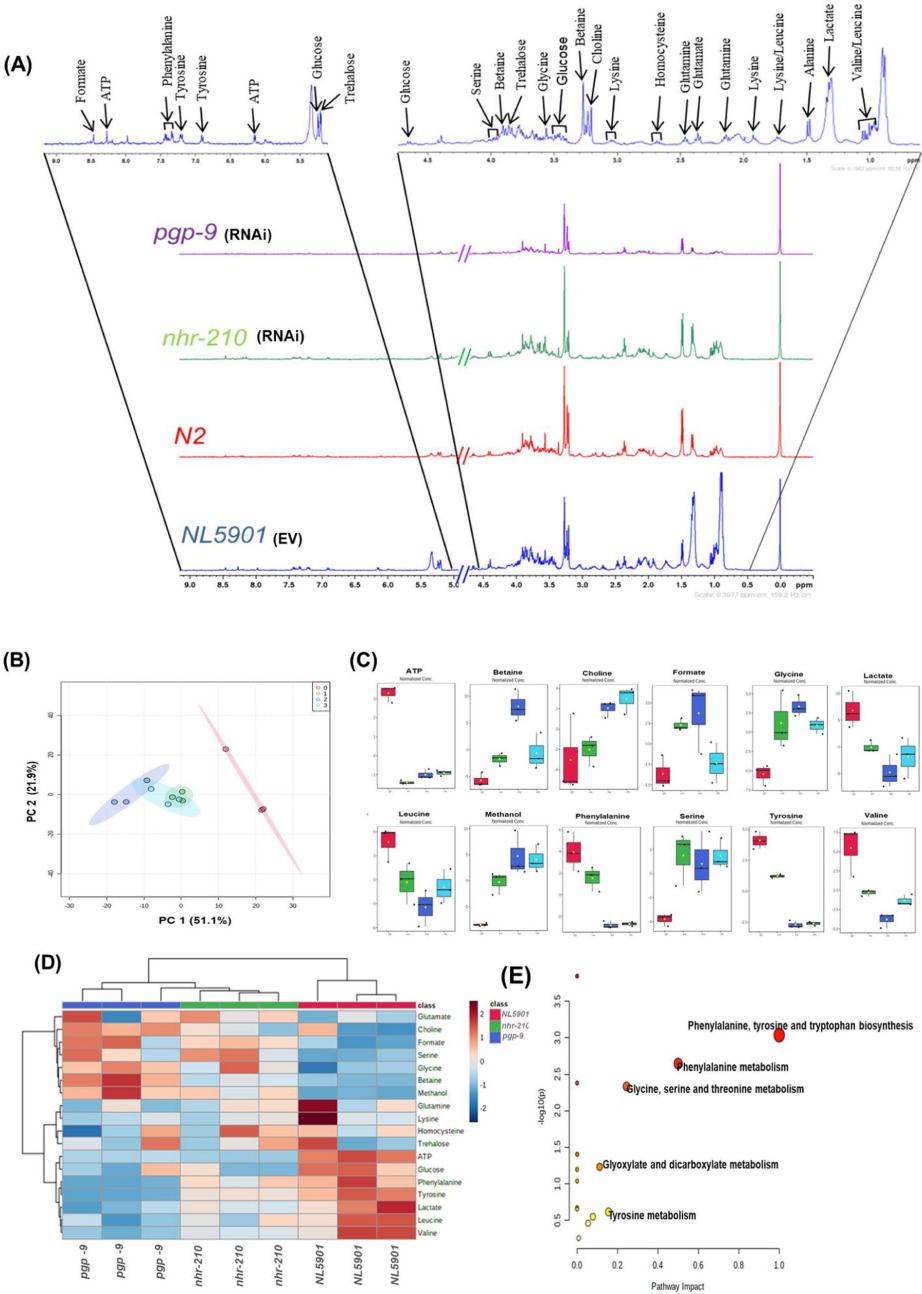
**A** Stack plot of HR-MAS ^1^H NMR CPMG spectrum of different worm samples with assigned metabolites (*n* = 10,000 worms). **B** 2D PCA score plots of worm samples; where Group-0, Group-1, Group-2 and Group-3 correspond to EV*, nhr-210*, *pgp-9* RNAi in NL5901 strain and N2 (wild type)*,* respectively. **C** Box-whiskers graphical representation of significantly different metabolites (*P* ≤ 0.05) where Group-0, Group-1, Group-2 and Group-3 correspond to RNAi of EV, *nhr-210*, *pgp-9* and N2 strain (wild type) respectively. **D** Heatmap plot for correlation of metabolites among different groups of worm samples. Columns represent groups, rows represent different metabolites and colour corresponds to their concentration (red corresponds to increase in concentration, while blue corresponds to decrease in concentration). **E** Pathway analysis of significant metabolites among worm sample

Metabolic assignment of *C. elegans* worm samples were performed in ^1^H CPMGPR1D NMR spectra as shown in Fig. 7A. Metabolites were unambiguously assigned and quantified by Chenomx profiler module. The online web based database i.e. Biological Magnetic Resonance Bank (BMRB, http://www.bmrb.wisc.edu) and Human Metabolome Database (HMDB, http://www.hmdb.ca) and reported literature were also used to confirm the identity of metabolites by comparing their chemical shift values, coupling constant and splitting pattern [31]. Assigned metabolites include ATP, alanine, acetate, betaine, glucose, glutamine, glutamate, glycine, inosine, lactate, leucine, lysine, methanol, phenylalanine, trehalose, tyrosine, and valine. Concentration of metabolites was express in terms of mean value ± standard deviation (Table 1).

**Table 1:** Concentration (mM) of significant metabolites observed in *C. elegans* worms

S. no	Metabolites	Concentration (mM)				*P*-value
		NL5901 (EV) (Mean ± SD))	NL5901 (KD *nhr-210*) (Mean ± sd)	NL5901 (KD *pgp-9)* (Mean ± sd)	*N2* (*wild type*) (Mean ± sd)	
1	ATP	0.1263 ± 0.018	–	–	–	0.000000070424
2	Betaine	0.3969 ± 0.125	0.7604 ± 0.169	1.0144 ± 0.308	0.5138 ± 0.164	0.00078185
3	Choline	0.1420 ± 0.161	0.1994 ± 0.054	0.2543 ± 0.046	0.2583 ± 0.049	0.027617
4	Formate	0.1732 ± 0.107	0.5085 ± 0.081	0.4113 ± 0.210	0.1583 ± 0.069	0.027613
5	Glycine	0.5452 ± 0.122	0.9857 ± 0.287	0.7284 ± 0.154	0.5868 ± 0.087	0.0062887
6	Lactate	1.4741 ± 0.423	1.2156 ± 0.066	0.5856 ± 0.041	0.6833 ± 0.239	0.0094196
7	Leucine	0.6125 ± 0.142	0.4627 ± 0.134	0.2092 ± 0.091	0.2691 ± 0.094	0.014971
8	Methanol	–	0.7555 ± 0.135	0.7933 ± 0.331	0.6709 ± 0.099	0.0013142
9	Phenylalanine	0.2909 ± 0.020	0.2442 ± 0.050	–	–	0.00013925
10	Serine	0.2622 ± 0.064	0.4955 ± 0.089	0.3232 ± 0.115	0.3020 ± 0.026	0.01918
11	Tyrosine	0.2789 ± 0.041	0.2112 ± 0.029	–	–	0.000000068561
12	Valine	0.6336 ± 0.172	0.4391 ± 0.040	0.1734 ± 0.038	0.2377 ± 0.051	0.0011021

Univariate (one-way ANOVA study) and multivariate statistical analysis (PCA and heatmap analysis) as well as pathway analysis were performed on concentration-profiled data, by employing metaboanalyst 5.0 (https://www.metaboanalyst.ca) an online web based server. Initially data was normalized and pareto scaled before multivariate analysis. Principal component analysis (PCA) was performed to identify the trend and the clustering pattern was observed among the groups in terms of principal components (PC), where PC1 and PC2 together explains 73% of the overall variance, while PC1 contributes 51.1% and PC2 contributes 21.9% of the explained variance (Fig. 7B). Further, one-way analysis of variance (ANOVA) was performed to determine the statistically significant metabolites. The significant metabolites having *P*-value ≤ 0.05 presented in box whiskers plot (Fig. 7C). Further, heatmap analysis provided an intuitive visualization of the metabolic change occurs in *nhr-210* and *pgp-9* with respect to EV (control) in NL5901 strain of *C. elegans* [32]. Furthermore, pathway analysis was performed on statistically significant metabolites [33] (Fig. 7D).

With reference to our previous results, we observed that *nhr-210* modulate worm behavior, and its predicted target *pgp-9* negatively regulates mitochondrial function. Therefore, it becomes intriguing to explore the role of *nhr-210* and *pgp-9* in the metabolic homeostasis, as there are reports that put forth the role of *nhr’s* as metabolic regulators [28]. Hence, we endeavor to answer this question through high-resolution magic angle spinning (HR-MAS) NMR spectroscopy. High-resolution magic angle spinning (HR-MAS) NMR is a reliable and reproducible technique for biological samples that can easily be applied on direct analysis of semi-solid, tissues and gel-like samples, etc., without any further extraction. In the present study, we decipher the variations in metabolites in *C. elegans* samples of group NL5901 strain with knockdown of EV, *nhr-210, pgp-9,* and N2 strain (wild type) as basal control. Our study revealed significant variation in key metabolites, which include ATP, betaine, choline, formate, glycine, lactate, leucine, methanol, phenylalanine, serine, tyrosine, and valine levels upon the knockdown of *nhr-210* and *pgp-9* with respect to control (EV) in the NL5901 strain of *C. elegans.* Moreover, we observe a particular trend in the levels of a few critical metabolites, such as levels of ATP, lactate, leucine, phenylalanine, tyrosine, and valine; showing reduced concentrations and an increase in concentration trend in betaine, choline, glycine, methanol, and serine upon the knockdown of *nhr-210* and *pgp-9* with respect control (EV) in NL5901 strain of *C. elegans*. As reported earlier, alteration of lactate and ATP occur due to mitochondrial dysfunction, which leads to various neurological disorders including Parkinson’s disease [34–36]. Interestingly, the significant decrease in the levels of ATP, suggests mitochondrial dysfunction. Glycine, reported to be an antioxidant agent and reduces oxidative stress markers in the infarcted regions in the brain [37], increases in groups *nhr-210* and *pgp-9* as compared with *NL5901* and reveals a response against oxidative imbalance*.* At the same time betaine, that shows neuroprotective effects [38], increases in concentration upon the knockdown of *nhr-210* and *pgp-9* with respect control (EV) in NL5901 strain of *C. elegans*. Furthermore, in the detailed examination of the global alteration of metabolic pathways upon the knockdown of *nhr-210* and *pgp-9*, we found that Phenylalanine, tyrosine and tryptophan biosynthesis metabolism; Glycine, serine, and threonine metabolism; Phenylalanine metabolism; Glycine, serine and threonine metabolism; Glyoxalate and dicarboxylate metabolism; and Tyrosine metabolism pathways are most altered (Fig. 7E). These pathways have been already reported to be associated with vast aliments of neurodegenerative diseases [39]. Conclusively, our results show a common trend upon the knockdown of *nhr-210* and *pgp-9*, therefore revealing close association with each other, followed by involvement in metabolic homeostasis.

## Discussion

Understanding age-related neurodegenerative diseases remains a significant challenge in research, largely due to their complex and multifactorial characteristics [40]. Traditionally, the emphasis in neurological research has predominantly been on the functioning and dysfunctions of neurons. However, recent years have witnessed a notable shift in focus. The scientific community is increasingly recognizing the importance of glial cells, a critical component of the neural ecosystem, in the functioning of the nervous system and the development of related diseases [41, 42]. Our study addresses this gap by delving into the role of nuclear hormone receptors (NHRs) that are enriched in *C. elegans* CEPsh glia, the counterparts of vertebrate astrocytes.

NHRs are transcription factors involved in diverse biological processes and have been identified as important players in neurodegeneration and neuroprotection [43–45]. We studied a range of CEPsh enriched genes, including *fmi-1, glt-1, nhr-210, nhr-231, clc-1,* and *ifa-4*. NHR-210 emerged as particularly intriguing due to its predicted transcription factor activity and potential intranuclear localization, a trait that may suggest its critical role in modulating gene expression. In our investigations, *nhr-210* emerged as a promising candidate due to its predicted transcription factor activity and its presumable location inside the nucleus. The potential relevance of *nhr-210* was substantiated by our observations that *nhr-210* knockdown in *C. elegans* alpha-synuclein expressing models (NL5901 expresses alpha synuclein in muscles and UA44 expresses alpha synuclein specifically in dopaminergic neurons) reduced α-synuclein aggregation, a hallmark of several neurodegenerative diseases [46], 47, alongside global ROS levels and influenced behavioral functions. Our study also unveiled the intriguing relationship between *nhr-210* and *pgp-9*, a target protein of *nhr-210*. *pgp-9* is an ATP efflux transmembrane transporter known for its role in drug resistance [47]. Intriguingly, we found that the expression of *nhr-210* markedly decreases in the presence of human alpha-synuclein aggregates. This reduction appears to trigger a compensatory increase in *pgp-9* expression, creating a feedback mechanism that helps the organism resist cellular death. Supporting this observation, the targeted reduction of *nhr-210* leads to elevated *pgp-9* transcript levels, indicating their involvement in a similar feedback process. Consequently, our findings imply that *nhr-210* may play a neuroprotective role, potentially through the upregulation of *pgp-9* expression. Our results propose *pgp-9* as a key regulator of active mitochondrial content and the stability of mitochondrial membrane potential in the NL5901 and UA44 strains of *C. elegans*. This is consistent with several studies establishing the critical role of mitochondrial dysfunction in neurodegenerative diseases [48–50]. The knockdown of *pgp-9* not only disrupted mitochondrial homeostasis but also induced significant cellular apoptosis. This observation expands on the well-established concept of mitochondrial dysfunction leading to programmed cell death [51–55] and suggests that *pgp-9* could be a pivotal player in the induction of mitochondrial-mediated apoptosis, a process implicated in neurodegeneration [56–58].

High-resolution magic angle spinning (HR-MAS) Nuclear Magnetic Resonance (NMR) based metabolomics, a robust tool for metabolic profiling, revealed alterations in key metabolic pathways, notably phenylalanine and tyrosine, precursors for dopamine synthesis. This links the dysregulation of mitochondrial functions to a potential disruption in dopamine synthesis, a neurotransmitter crucially implicated in several neurodegenerative diseases, particularly Parkinson's disease [56–58].

Our findings uncover a potential link between *nhr-210* and *pgp-9* dysregulation, mitochondrial dysfunction, and perturbations in dopamine synthesis, which aligns with the widely recognized role of mitochondrial damage and dopamine metabolism in the pathogenesis of Parkinson's disease [56–58]. Consequently, our work also suggests the possible therapeutic potential of targeting the *nhr-210/pgp-9* pathway to maintain mitochondrial health, sustain metabolic homeostasis, and possibly slow the progression of neurodegenerative diseases.

In summary, our study uncovers the complex interplay between CEPsh glia-enriched *nhr-210* and *pgp-9*, elucidating their potential role in mitochondrial function, apoptosis, and metabolic homeostasis. The findings also provide insights into the relevance of *nhr-210* abundance within the CEPsh glia. We propose that *nhr-210* modulates *pgp-9*, which subsequently affects cellular apoptosis and contributes to the underlying mechanisms of neurodegeneration. This critical crosstalk could likely contribute to the modulation of metabolic homeostasis and trigger the shift towards pathophysiological states, as observed in neurodegenerative diseases.

## Data Availability

The data that support the findings of this study are available from the corresponding author upon reasonable request.

## Abbreviations

CEPsh: CEPhalic sheath glia
NHR’s: Nuclear hormone receptors
HR-MAS NMR: High-resolution magic angle spinning NMR spectroscopy
RNAi: RNA interference
